# CGG Repeat Expansion, and Elevated *Fmr1* Transcription and Mitochondrial Copy Number in a New Fragile X PM Mouse Embryonic Stem Cell Model

**DOI:** 10.3389/fcell.2020.00482

**Published:** 2020-06-30

**Authors:** Inbal Gazy, Carson J. Miller, Geum-Yi Kim, Karen Usdin

**Affiliations:** ^1^Laboratory of Cell and Molecular Biology, NIDDK, National Institutes of Health, Bethesda, MD, United States; ^2^KwaZulu-Natal Research Innovation and Sequencing Platform (KRISP), College of Health Sciences, University of KwaZulu-Natal, Durban, South Africa

**Keywords:** repeat expansion, Fragile X syndrome, Fragile X-related disorders, premutation, mouse embryonic stem cells, mitochondrial abnormalities, *Fmr1* hyperexpression

## Abstract

The Fragile-X related disorders (FXDs) are Repeat Expansion Diseases (REDs) that result from expansion of a CGG-repeat tract located at the 5′ end of the *FMR1* gene. While expansion affects transmission risk and can also affect disease risk and severity, the underlying molecular mechanism responsible is unknown. Despite the fact that expanded alleles can be seen both in humans and mouse models *in vivo*, existing patient-derived cells do not show significant repeat expansions even after extended periods in culture. In order to develop a good tissue culture model for studying expansions we tested whether mouse embryonic stem cells (mESCs) carrying an expanded CGG repeat tract in the endogenous *Fmr1* gene are permissive for expansion. We show here that these mESCs have a very high frequency of expansion that allows changes in the repeat number to be seen within a matter of days. CRISPR-Cas9 gene editing of these cells suggests that this may be due in part to the fact that non-homologous end-joining (NHEJ), which is able to protect against expansions in some cell types, is not effective in mESCs. CRISPR-Cas9 gene editing also shows that these expansions are MSH2-dependent, consistent with those seen *in vivo*. While comparable human Genome Wide Association (GWA) studies are not available for the FXDs, such studies have implicated MSH2 in expansion in other REDs. The shared unusual requirement for MSH2 for this type of microsatellite instability suggests that this new cell-based system is relevant for understanding the mechanism responsible for this peculiar type of mutation in humans. The high frequency of expansions and the ease of gene editing these cells should expedite the identification of factors that affect expansion risk. Additionally, we found that, as with cells from human premutation (PM) carriers, these cell lines have elevated mitochondrial copy numbers and *Fmr1* hyperexpression, that we show here is O_2_-sensitive. Thus, this new stem cell model should facilitate studies of both repeat expansion and the consequences of expansion during early embryonic development.

## Introduction

The Fragile X-related disorders (FXDs), are members of the Repeat Expansion Disorders (REDs), a group of 35+ human diseases that arise due to an expansion or increase in the length of a disease-specific microsatellite. The microsatellite responsible for the FXDs is a CGG repeat tract located in the 5′ untranslated region (UTR) of the X-linked *FMR1* gene. Premutation (PM) alleles contain 55–200 repeats and carriers of such alleles are at risk of developing Fragile X-associated tremor/ataxia syndrome (FXTAS), a late-onset neurodegenerative disorder, and Fragile X-associated primary ovarian insufficiency (FXPOI), a cause of early menopause and infertility of women carriers before the age of 40 (Mila et al., 2018). In addition to the risk of FXTAS and FXPOI, women carrying a PM allele are at risk of transmitting a much larger full mutation (FM) allele to their children. Such alleles contain more than 200 repeats and give rise to a third disorder, Fragile X syndrome, the leading cause of inherited intellectual disability and autism spectrum disorder (ASD; Crawford et al., 2001).

Expansions into the PM range result in elevated *FMR1* transcript levels (Tassone et al., 2000). However, this increase in transcript levels does not result in increased production of FMRP, the protein product of *FMR1*. In fact, FMRP levels are reduced in PM cells due to impaired translation of transcripts with large numbers of CGG repeats (Kenneson et al., 2001). PM pathology results from the deleterious consequences of the PM transcripts (Renoux and Todd, 2012) that are likely to be exacerbated by the elevated levels of the expanded CGG-repeat containing *FMR1* transcript. While expansions have important consequences for disease pathology in humans, the underlying mechanism responsible for the expansion mutation is still largely unclear, as is the timing of the expansion from a PM to a FM allele.

Unlike other REDs where patient derived cell-culture models show a progressive increase in repeat number over time in culture (Cannella et al., 2009; Du et al., 2012, 2013), expansion in differentiated cells from human FX PM carriers, induced pluripotent stem cells (iPSC) derived from these cells, or human embryonic stem cells (hESCs) carrying large unmethylated alleles, either does not occur or occurs extremely rarely (Brykczynska et al., 2016; Zhou et al., 2016). Furthermore, expansion in a PM knock-in (KI) mouse model (Entezam et al., 2007) is known to be very cell type specific (Lokanga et al., 2013; Zhao and Usdin, 2018; Gazy et al., 2019). Thus, in order to develop a good tissue culture model for repeat expansion that could be used to expedite studies of the expansion mechanism, we needed to identify a cell type permissive for this mutation that can be readily cultured for long periods. Work with PM mice suggested that expansions occur at a high frequency in the early embryo (Lokanga et al., 2014; Zhao et al., 2016). Since expansions are not seen in hESCs (Zhou et al., 2016), a cell type that is thought to resemble the more developmentally advanced primed EpiSCs rather than naïve ESCs (Smith, 2017), we hypothesized that high frequency expansions in the early embryo may be limited to cells more reminiscent of earlier stages in embryonic development. Given that mouse embryonic stem cells (mESCs) have the characteristics of more naïve ESCs (Smith, 2017), it was possible that mESCs generated from PM mice would show expansions that occur at high enough frequency to allow the expansion process to be studied *in vitro*. We show here that indeed this is the case, with expansions occurring in most cells in the mESC population as often as twice a week depending on the repeat number. This very high mutation rate has implications for the mechanism involved. It also allows factors that affect this mutation rate to be readily examined. The PM mESC lines also display cellular changes that resemble those found in cells of human PM carriers. Thus, these mESCs can serve as a useful model to facilitate our understanding both of the expansion mechanism and its consequences.

## Materials and Methods

### Generation of mESCs Lines

The C57BL/6 *Fmr1* FX KI (knock-in) mice were described previously (Entezam et al., 2007). Mice were maintained in accordance with the guidelines of the NIDDK Animal Care and Use Committee and with the Guide for the Care and Use of Laboratory Animals (NIH publication no. 85–23, revised 1996). Embryos were isolated from superovulated *Fmr1*^*WT/KI*^ females mated with *Fmr1*^*WT*^ males. Preimplantation embryos were obtained by flushing the uterine horns with M2 medium (GSM-5120, MTI-GlobalStem, Rockville, MD, United States) at post coitum day 3.5. Embryos were first washed with M2, then a 1:1 mixture of M2 and KSOM medium (GSM-5140, MTI-GlobalStem), and finally with KSOM. Embryos were then plated separately in 0.1% gelatin (ES-006-B, MilliporeSigma, St. Louis, MO, United States) coated wells pre-equilibrated with KSOM medium. Most embryos hatched from the zona pellucida 1–2 days after plating, at which point media was exchanged to N2B27 medium supplemented with 2i [3 μM CHIR99021 (S2924, Selleckchem, Houston, TX, United States), 1 μM PD0325901 (S1036, Selleckchem)], and LIF (1000 unit/ml; ESG1107, MilliporeSigma; hereafter referred to as N2B27 2i/LIF medium; Nichols and Ying, 2006; Ying et al., 2008). The embryos were grown for ∼7 days with daily medium changes until the emergence of ES-cell colonies. The cells were then trypsinized (TrypLE^TM^ Select, Thermo Fisher Scientific, Waltham, MA, United States) and transferred to new wells containing N2B27 2i/LIF supplemented with 10 ng/ml BMP-4 (314-BP-010, R&D Systems, Minneapolis, MN, United States). Once these cultures were ready for passaging, cells were replated and 1 day after plating the medium was replaced with N2B27 2i/LIF lacking BMP-4. For routine propagation, cells were maintained on 0.1% gelatin coated wells in N2B27 2i/LIF media (Nichols and Ying, 2006; Ying et al., 2008) with daily media changes and passaged 1:3–1:6 every 2–3 days. Evaluation of pluripotency markers was carried out using standard immunofluorence protocols. Briefly, cells were fixed in 4% PFA, permeabilized, and blocked with 0.3% Triton X-100, 10% normal goat serum in PBS prior to immunostaining. Details of primary antibodies used are provided in Supplementary Table S4. Primary antibodies were detected with appropriate secondary antibodies labeled with Alexa-Fluor 555 (Thermo Fisher Scientific). Images were acquired using EVOS FL Microscope (Thermo Fisher Scientific).

### Generation of *Msh2*^–^*^/^*^–^, *Lig4*^–^*^/^*^–^, and *Pkrdc*^–^*^/^*^–^ mESCs

For each gene to be edited 2 guide RNAs (gRNAs) were used in conjunction with a single-stranded oligonucleotide (ssODN) and CRISPR-Cas9 to generate null lines. The gRNAs and ssODNs are listed in Supplementary Table S1. The relevant DNAs encoding the appropriate gRNAs were cloned into a modified pX459 V2.0 [a gift from Feng Zhang (Addgene plasmid # 62988; http://n2t.net/addgene:62988; and RRID:Addgene_62988; (Ran et al., 2013)] with each gRNA expressed under a human U6 promoter and containing a downstream gRNA scaffold. Transfection of a mESC line containing ∼170 repeats was carried out using Lipofectamine^®^ LTX reagent with PLUS^TM^ reagent (15338030, Thermo Fisher Scientific) following the manufacturer’s protocol. Control lines were obtained by mock transfections without a gRNA construct. To mitigate potential off-target effects we used multiple independently derived cell lines for these experiments. Three micrograms of the gRNAs/Cas9 expressing plasmids, 1 μg ssODN, and 4 μl PLUS^TM^ reagent were mixed into 250 μl OPTI-MEM (Thermo Fisher Scientific) and incubated for 5 min at room temperature. 12 μl of Lipofectamine LTX reagent was diluted into 240 μl of OPTI-MEM and combined with the DNA:PLUS^TM^ mixture and incubated for 30 min at room temperature. Two hundred thousand mESC cells were suspended in 1 ml N2B27 2i/LIF and mixed with the DNA:PLUS^TM^:Lipofectamine LTX mixture and plated onto a well of a 6-well plate. Cells were incubated with the transfection mix for 4–6 h at 37°C. Transfected cells were collected, pelleted and resuspended in fresh media and plated in a 60 mm dish. 24 h post transfection, cells were selected with puromycin (1 μg/ml) for 24 h, then grown for 4–5 more days. Single colonies were picked and plated in a 24-well plate. DNA was isolated from established clones and analyzed using PCR amplification and sequencing of either the PCR products directly or cloned products. The primers used are listed in Supplementary Table S2. The sequences of the relevant alleles in the mutant lines chosen for further analysis are shown in Supplementary Figure S2. All mutations involved large deletions. The loss of protein expression in these lines was then verified using western blots of total proteins extracted from these cells using standard procedures (Supplementary Figure S3). No significant differences were noted in the growth rates of any of these cell lines. For the evaluation of the effects of the mutations on repeat instability we picked control and mutant cell lines that were match for repeat number. The repeat number of the cell lines is shown in the relevant figures. For all experiments, cells were used at early passage numbers to minimize any potential selection artifacts.

### Analysis of Repeat Expansions

Control and mutant cell lines matched for repeat number were grown in culture for the indicated number of days. Cells were passaged every third day by treatment with TrypLE^TM^ Select according to the supplier’s instructions, followed by inactivation by growth medium containing 15% ES qualified FBS (Sigma-Aldrich, St. Louis, MO, United States). The cell suspension was replated ∼1:6 into 0.1% gelatin coated wells for further propagation while DNA was made from the remaining cells. DNA isolation was carried out by resuspending cells in lysis buffer (10 mM Tris–HCl pH 7.5, 400 mM NaCl, 100 mM EDTA pH 8.0, and 0.6% SDS) with 0.58 mg/ml proteinase K solution (Thermo Fisher Scientific), and incubating the cell suspension at 55°C overnight before the addition of 1.25 M NaCl. The resultant precipitate was pelleted by centrifugation and equal volume of 100% ethanol was added to supernatant. DNA was pelleted and dissolved in TE. All genotyping PCRs were carried out using KAPA2G Fast HotStart Genotyping Mix (KK5621, KAPA Biosystems, Wilmington, MA, United States) according to the manufacturer’s instructions. *Fmr1* PM allele genotyping and repeat number evaluation was performed on bulk DNA using a fluorescent PCR assay with a FAM-labeled FraxM4 and unlabeled FraxM5 primer pair (Supplementary Table S2). PCR was carried out using the KAPA2G Fast HotStart Genotyping Mix supplemented with 2.5 M betaine (Sigma-Aldrich), 2% DMSO (americanBIO, Natick, MA, United States), and 0.12 mM dGTP and dCTP (Thermo Scientific), and PCR parameters: 95°C for 10 min, 35 × (95°C for 15 s, 65°C for 15 s, and 72°C for 30 s), 72°C for 10 min. Small pool PCR (SP-PCR) was carried out on diluted DNA using nested PCR. The first round of PCR was carried out using FraxC and FraxF primer pair (Supplementary Table S2). PCR was carried out using the KAPA2G Fast HotStart Genotyping Mix supplemented with 2.5 M betaine (Sigma-Aldrich), 2% DMSO, and PCR parameters: 95°C for 10 min, 30 × (95°C for 15 s, 65°C for 15 s, and 72°C for 30 s), 72°C for 10 min. One microliter of this PCR mix was used in second round of PCR with FAM-labeled FraxM4 and FraxM5 primer pair (Supplementary Table S2). PCR was carried out using the KAPA2G Fast HotStart Genotyping Mix supplemented with 2.5 M betaine (Sigma-Aldrich, St. Louis, MO, United States), 2% DMSO and 0.12 mM dGTP and dCTP (Thermo scientific, Waltham, MA, United States), and PCR parameters: 95°C for 5 min, 30 × (95°C for 15 s, 65°C for 15 s, and 72°C for 30 s), 72°C for 10 min. Repeat PCR reactions were resolved by capillary electrophoresis on an ABI Genetic Analyzer (Roy J Carver Biotechnology Center, University of Illinois, Urbana, IL, United States). The resultant fsa file was then displayed using a custom R script that is available upon request (Hayward et al., 2016).

### RNA Isolation and Quantitative RT−PCR (qRT-PCR)

RNA was isolated from cell lines with 8 repeats wild-type (WT) and from lines with ∼170 repeats (PM) using TRIzol^TM^ reagent, treated with DNase and reverse transcribed with SuperScript IV VILO Master Mix with ezDNase Enzyme (all from Thermo Fisher Scientific). Transcript levels were determined by qPCR of the resultant cDNA using the TaqMan assays listed in Supplementary Table S3. Since an examination of GEO Datasets^1^ of RNA-seq data from neurons in *Fmr1* WT and KO mice [(Korb et al., 2017): GSE81803]; [(Ding et al., 2020): GSE114015]; and microarray data comparing gene expression differences in the blood of human carriers of normal and PM alleles [(Mateu-Huertas et al., 2014): GSE48873] showed no significant difference in β-actin levels, β-actin was used for normalization.

### DNA-RNA Immunoprecipitation (DRIP) Assay

DNA-RNA immunoprecipitation assays were performed on cells containing either 8 repeats (WT) or ∼170 repeats (PM) as described previously (Kumari and Usdin, 2016) with slight modifications. For each DNA sample, three DRIP assays were performed: a no antibody control and assays with S9.6 antibody either without or with RNAse H pretreatment. A total of 25 μg DNA was either mock digested or digested with 1.7 units of RNAse H (M0297S, New England Biolabs, Ipswich, MA, United States) per 1 μg DNA in 100 μl final volume at 37°C for 6 h. Three hundred microliters of ChIP dilution buffer (167 mM NaCl, 0.01% SDS, 1.1% Triton X-100, 1.2 mM EDTA, and 16.7 mM Tris, pH 8.0) was added to each sample and the samples were then sonicated using the medium setting on the Bioruptor sonication system (Diagenode, Denville, NJ, United States) with cycles of 30 s on/30 s off for 10 min. To 350 μl of the sonicated DNA, 650 μl of ChIP dilution buffer supplemented with protease inhibitor cocktail (P8340-5ML, Sigma-Aldrich) was added and mixed. An aliquot (1%) was saved as input sample. The sonicated DNA was then precleared with 50 μl of Protein A agarose beads/Salmon sperm DNA slurry (16–157, EMD MilliporeSigma) for 1 h on a rotator at 4°C. The precleared supernatant was incubated with or without 5 μg S9.6 antibody (MABE1095, EMD MilliporeSigma) overnight on a rotator at 4°C. The sample was then incubated with 60 μl of the Protein A agarose beads/salmon sperm DNA slurry for 1 h on a rotator at 4°C to collect the immune complexes. The material was washed with low-salt washing buffer (150 mM NaCl, 0.1% SDS, 1% Triton X-100, 2 mM EDTA, and 20 mM Tris–HCl, pH 8.0) followed by high-salt washing buffer (500 mM NaCl, 0.1% SDS, 1% Triton X-100, 2 mM EDTA, and 20 mM Tris–HCl, pH 8.0), LiCl immune complex wash buffer [0.25 M LiCl, 1% IGEPAL CA630, 1% deoxycholic acid (sodium salt), 1 mM EDTA, 10 mM Tris, pH 8.0], and finally washed twice with TE buffer. The immunoprecipitated material was then eluted from the beads using elution buffer (1% SDS and 0.1 M NaHCO_3_). The input and DRIP samples were treated with phenol/chloroform and precipitated overnight at −20°C with 120 mM sodium acetate and ethanol. After washing with 70% ethanol, the samples were resuspended in 50 μl 0.1X TE, pH 8.0. Real-time PCR was carried out using the PowerUp^TM^ SYBR^®^ Green Master Mix (A25778, Thermo Fisher Scientific) and a StepOnePlus Real-Time PCR system (Thermo Fisher Scientific). β*-actin* was used as a positive control (Skourti-Stathaki et al., 2019). The primer sequences are provided in Supplementary Table S2.

### Evaluation of Mitochondrial DNA Copy Number

The relative mitochondrial DNA (mtDNA) copy number was measured in cell lines containing either 8 repeats (WT) or ∼170 repeats (PM) using real-time PCR to determine the levels of a mitochondrial gene, *COXI*, relative to a nuclear gene, *GAPDH*, using the primer pairs shown in Supplementary Table S2. The relative mtDNA copy number was calculated using the 2^–ΔΔCt^ method. Primer sequences are provided in Supplementary Table S2.

### Western Blotting

Cell pellets from cells with either 8 repeats WT or ∼170 repeats PM were collected in RIPA buffer (150 mM NaCl, 1.0% NP-40, 0.5% DOC, 0.1% SDS, 50 mm Tris pH 8.0, and protease inhibitor cocktail) and sonicated at 40% power 5 times 10 s on/10 s off using a Branson 250 Sonifier. Proteins (30–50 μg) were separated on polyacrylamide gels (Thermo Fisher Scientific) and transferred onto nitrocellulose membrane (Bio-Rad Laboratories, Hercules, CA, United States). Primary antibodies used are listed in Supplementary Table S4. Images were taken with ChemiDoc imaging system (Bio-Rad Laboratories).

### Statistical Analysis

Statistical analysis of the SP-PCR data was carried out using the Mann-Whitney *U* test^2^. Other comparisons were based on Student’s *t* test using an unpaired, two-tailed distribution.

## Results

### mESCs With an Expanded CGG Tract in the *Fmr1* Gene Show a High Frequency of MSH2-Dependent Repeat Expansions *in vitro*

We derived male mESC lines from pre-implantation embryos of *Fmr1*^*WT/KI*^ females carrying different numbers of repeats, as described in the section “Materials and Methods.” Lines containing 130, 182, and 292 repeats were initially chosen for further study. We included a line with an allele in the FM range since mice with FM sized alleles do not become methylated (Entezam et al., 2007). Thus, the allele with 292 repeats should still be capable of expansion in a permissive cell type. The derived KI mESC lines displayed similar morphology and expressed pluripotency factors at the same levels as WT control lines carrying the normal murine *Fmr1* allele (Supplementary Figures S1A,B).

**FIGURE 1:**
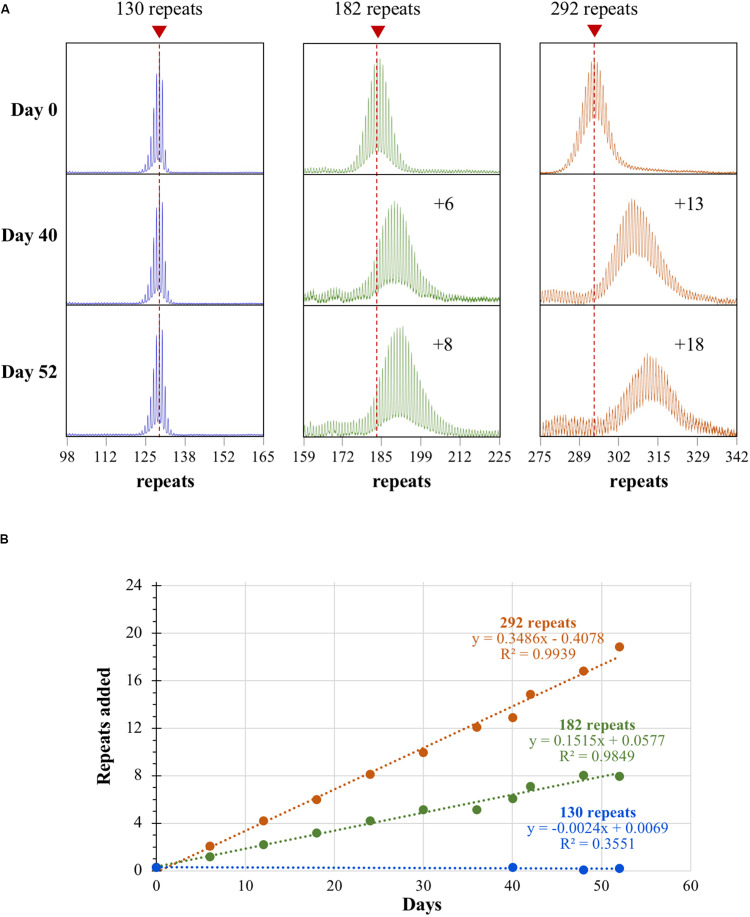
Repeat expansion in mESCs with different numbers of CGG repeats. **(A)** Repeat PCR profiles for cell lines with 130, 182, and 292 repeats after 0, 40, and 52 days in culture. Dashed lines represent the initial allele size at day 0. **(B)** Graph showing the change in repeat number over time for the 3 mESC lines shown in **(A)**.

Repeat PCR analysis of the smallest cell line tested, one having 130 repeats, showed an initial PCR profile that had a left-skew, a characteristic we have previously shown to be associated with alleles that do not expand *in vivo* [(Figure 1A; Zhao et al., 2019)]. These cell lines show no significant increase in repeat number after 52 days in culture and no change in the skewness of the PCR profile. In contrast, lines with larger alleles start off, even at very low passage number, with a more normal allele distribution profile (Figure 1A). They also show a steady increase in the average repeat number over time such that the whole population of alleles shifts in an apparently synchronous fashion. The net effect being that the 182 repeat line gains on average 8 repeats over a 52-day period whilst the 292 repeat line gains 18 repeats (Figure 1A). Thus, mESCs indeed show frequent, length-dependent expansions. The failure to see expansion in the 130 repeat line is consistent with observations from mice and likely reflects a much slower expansion rate than is seen in the cell lines with larger repeat tracts. The repeat PCR profile for both cell lines containing larger repeat tracts is similar to what is seen in somatic cells from human PM carriers (Zhao et al., 2019) and in iPSCs from individuals with myotonic dystrophy type 1, another RED that shows large, maternally transmitted intergenerational expansions (Du et al., 2013). Computer simulations that are consistent with this sort of expansion profile require a high frequency of expansion events that add only 1–2 repeats with each event (Mollersen et al., 2010). Frequent sampling from the same mESC culture over time that is possible with the mESCs confirms this interpretation. These cells show sequential expansion events, each involving a gain of a single repeat, as can be seen in the line with 182 repeats, with an average of 1 repeat gained every ∼6 days which occurs in the bulk of cells in the population (Figure 1B). By way of comparison, a gain of an average of 8 repeats is seen in the brains of 6-month-old mice with a similar repeat number (Zhao and Usdin, unpublished observations). This corresponds to a ∼3.5-fold higher rate of expansion in mESCs. Thus, while most expansions are small, they can be so frequent in certain cell types such as mESCs that large alleles could readily arise over time from the cumulative effect of multiple high frequency expansion events.

**FIGURE 2:**
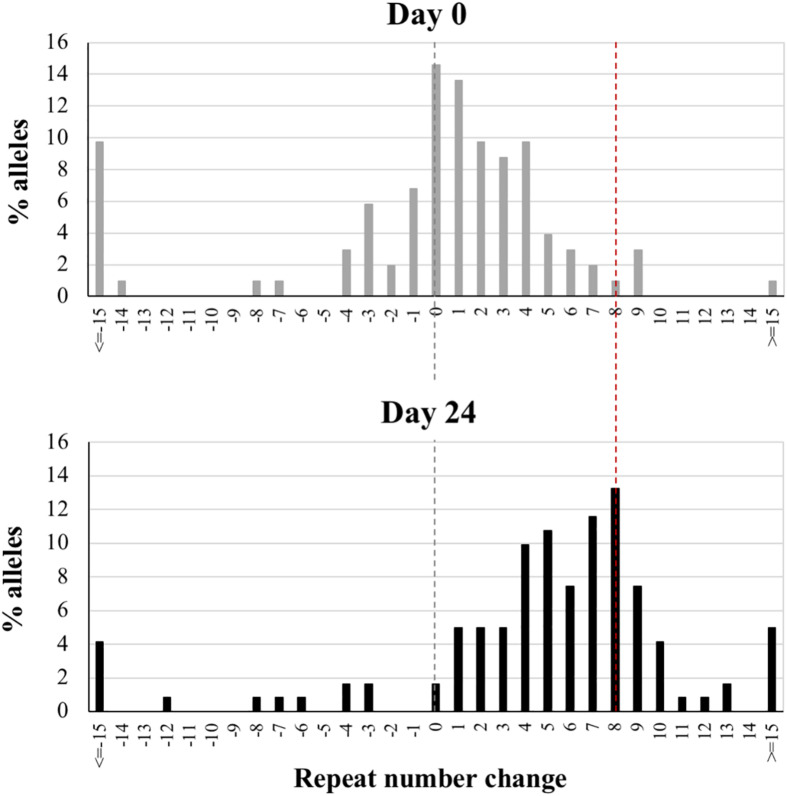
Small pool PCR analysis of PM cell line showing the change in the distribution of alleles with time. Nested PCR on diluted DNA from a cell line carrying 175 repeats was performed at day 0 and day 24. The nested PCR results in a ∼3 repeat decrease in the average PCR product relative to the bulk DNA because of the bias generated by strand-slippage during the PCR reactions. Thus, the distributions were corrected accordingly and the major allele at day 0 was set at 0 with the remaining alleles shown as the change in repeat number relative to this allele. The black dotted line reflects the major allele in the starting population and the red dotted line the major allele in the sample at 24 days. More than 100 alleles were examined for each sample.

Bulk PCR tends to bias the analysis toward the most common alleles. As a result, contractions and expansions that generate unique allele sizes are difficult to discern. In addition, the major peak in the bulk PCR profile represents a mixture of PCR stutter (resulting from strand-slippage during the PCR reaction across the repeats) and true peaks from multiple alleles. SP-PCR performed on single alleles can give a better representation of the true allele distribution. We therefore carried out SP-PCR on an mESC line carrying a mid-size PM allele (175 repeats) at day 0 (representing passage 10 after derivation) and day 24. As can be seen in Figure 2, SP-PCR demonstrates a strong shift in the distribution of alleles toward larger sizes at day 24, with the modal repeat number being 8 repeats larger than the modal repeat number at day 0. This is associated with a significant change in the distribution of allele sizes as assessed by the Mann-Whitney *U* test (*p* < 0.0001). The allele distributions seen in these cells are very similar to the distributions seen in the tissues of mice with a similar number of repeats (Zhao et al., 2016). Thus, both small expansions and large expansions occur in the PM mESCs, with small expansions predominating.

Large contractions are also seen, although at lower frequency. In some cases, cells containing specific contracted alleles tend to become more prominent over time. This likely reflects the difficulty associated with replication of long CGG-repeat tracts (Voineagu et al., 2008) resulting in a selective advantage for cells containing smaller alleles.

**FIGURE 3:**
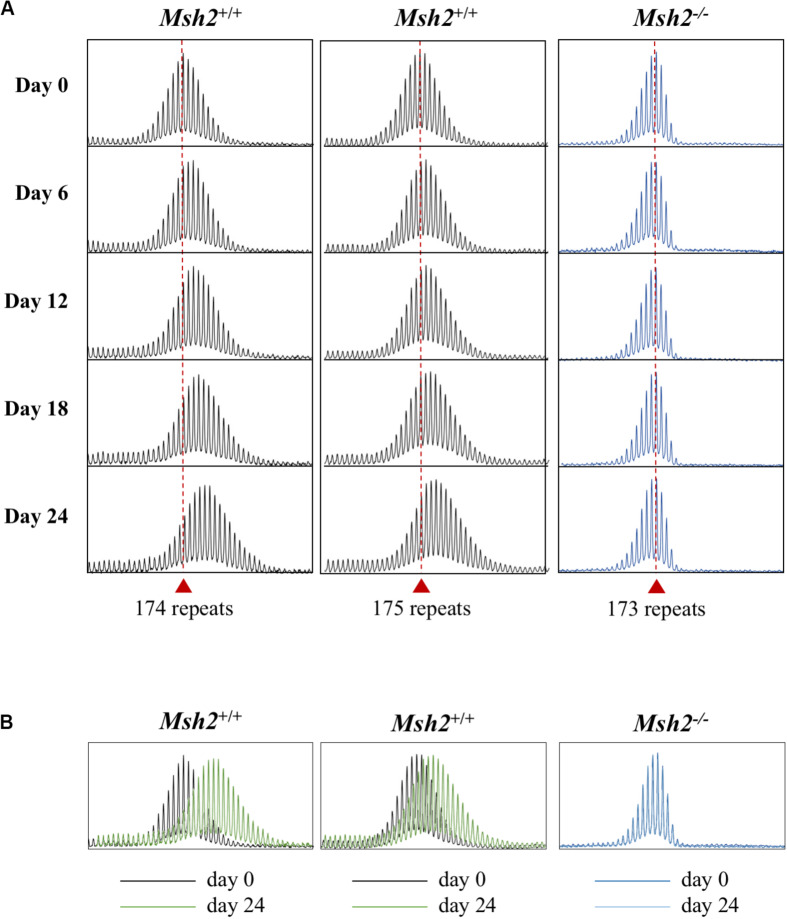
Effect of the loss of MSH2 on repeat expansion in mESCs. **(A)** Representative repeat PCR profiles of the 2 *Msh2*^+/+^ and 1 *Msh2^–/–^* lines with 173–175 repeats grown in culture for the indicated times. Dashed lines represent the initial allele at day 0. At least 2 mESC lines were tested for each genotype with similar results being obtained for each line. **(B)** Overlays of the day 0 and day 24 scans for each of the cell lines.

MSH2, a DNA mismatch repair protein, is essential for expansions in the FX KI mouse model (Lokanga et al., 2014), and Genome Wide Association (GWA) studies have implicated MSH2-containing complexes in the expansion process in other REDs (Bettencourt et al., 2016; Morales et al., 2016; Moss et al., 2017; Flower et al., 2019). Using CRISPR-Cas9-mediated gene disruption, we generated *Msh2* knockout PM mESC lines (Supplementary Figures S2A, S3A) and tested them in culture with size matched *Msh2*^+/+^ mESC lines. As can be seen in Figure 3, at day 0 *Msh*2^–/–^ cells show an allele profile with left-skew characteristic of cells that do not expand [(Figure 1A and (Zhao et al., 2019)]. After growth for 24 days, no repeats were added to the PM allele in these cells and no change in the profile skewness was observed. In contrast, *Msh2*^+/+^ cells show a more normal distribution of allele sizes and show clear evidence of expansion by day 24. This can be visualized clearly by comparing an overlay of the bulk PCR profiles from day 0 and day 24 for each cell line. The *Msh2*^+/+^ cells show a clear shift in the allele distribution, while the PCR profiles from *Msh*2^–/–^ cells are indistinguishable (Figure 3B). Thus, our data demonstrate that the PM mESC lines show CGG repeat expansion that has an MSH2-dependence and similar dynamics to those observed in mice and humans. This, together with their high expansion frequency, suggest that these PM mESCs are a useful system for studying expansion in the FXDs.

**FIGURE 4:**
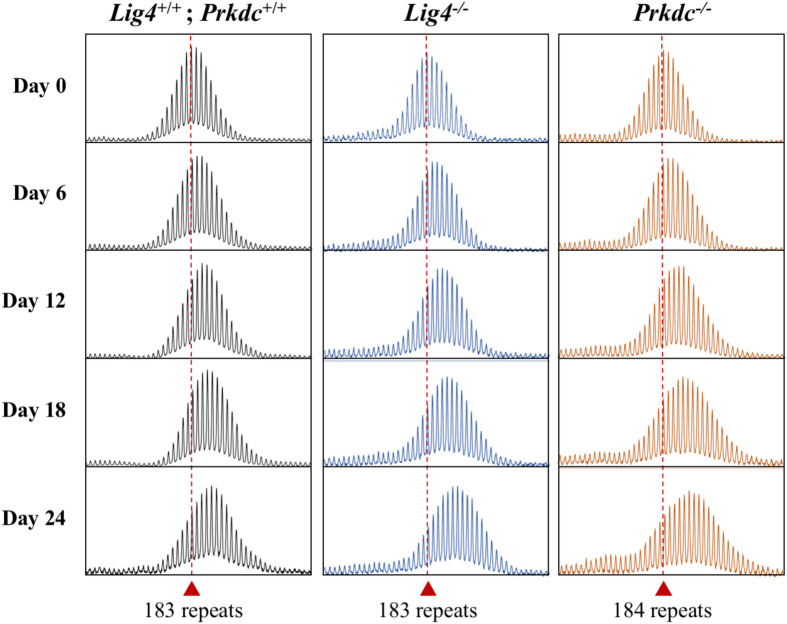
Effect of the loss of NHEJ proteins on repeat expansion in mESCs. Representative repeat PCR profiles of *Lig4*^+/+^; *Prkdc*^+/+^, *Lig4*^–/–^, and *Prkdc*^–/–^ mESCs with 183–184 repeats that were grown in culture for the indicated times. Dashed lines represent the initial repeat size at day 0. At least 2 mESC lines were tested for each genotype with similar results being obtained for each line.

We had previously shown that in liver LIG4, a DNA ligase essential for non-homologous end-joining (NHEJ), a form of double-strand break repair, protects against expansion (Gazy et al., 2019). We were unable to examine the contribution of LIG4 to embryonic expansion in these animals since the absence of LIG4 results in early embryonic lethality due to defective neurogenesis (Barnes et al., 1998). To investigate the role of NHEJ in embryonic cells we were now able to generate PM mESCs lines deficient for *Lig4* and *Prkdc*, which encodes the catalytic subunit of DNA-PK, another important NHEJ protein (Supplementary Figures S2B,C, S3B), as these lines are both viable. While loss of LIG4 in mouse livers results in a significant increase in expansions, the loss of LIG4, or DNA-PK resulted in expansions that were indistinguishable from those seen in WT cells (Figure 4). This is consistent with the fact that NHEJ is known to be less active in stem cells than in differentiated cells (Tichy et al., 2010). Thus, NHEJ has little, if any, protective effect against repeat expansion in the early embryo and this may account, at least in part, for the large number of expansions seen in these cells.

### *Fmr1* mRNA Levels Are Elevated in PM mESCs

**FIGURE 5:**
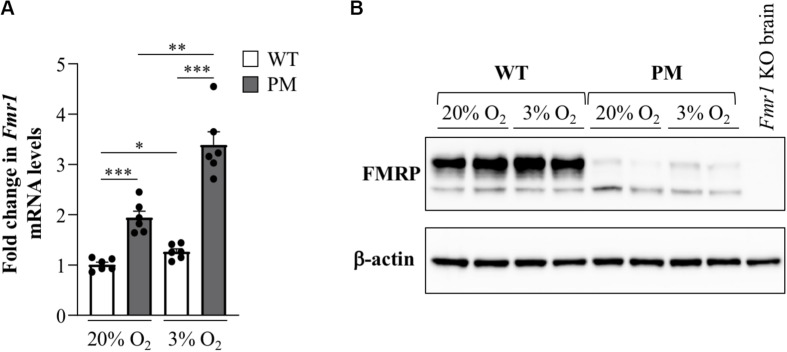
*Fmr1* expression in FX PM mESCs. **(A)** Quantitative reverse-transcription-PCR analysis of *Fmr1* expression in WT and PM lines cultured at 20% or 3% O_2_ using β*-actin* for normalization. Values are relative to the average expression of the WT lines cultured at 20% O_2_. Results are from 3 mESC lines for each condition and represented as mean ± SEM. *n* = 6; *p < 0.01; **p < 0.001; and ***p ≤ 0.00001. **(B)** Western blot analysis of FMRP expression in 2 WT and 2 PM lines cultured at 20% or 3% O_2_.

*FMR1* transcription is elevated in cells isolated from PM human carriers (Tassone et al., 2000) and in various tissues isolated from PM mice (Lokanga et al., 2013). To assess whether the same is true for mESCs, we measured *Fmr1* transcript levels in WT and PM mESCs with ∼170 repeats. It should be noted that mouse WT alleles only have 8 repeats, whilst most normal *FMR1* alleles in humans have ∼30 repeats. Whilst data from human studies suggest that alleles of different sizes within the normal range have similar transcript levels (Tassone et al., 2000), it is possible that any differences observed between the behavior of WT and PM alleles in these experiments, as well as those described in subsequent sections, might be larger than that expected in humans. However, we found only a 1.9-fold higher level of *Fmr1* mRNA in the PM lines compared to WT lines (Figure 5A). While significant, this difference is smaller than the typical differences seen in tissues of these animals as well as in human PM cells. Since cells in culture are typically grown at atmospheric O_2_ levels and the physiological O_2_ level in tissues and cells is much lower, between 1–9% depending on the tissue (Mas-Bargues et al., 2019), we tested the transcript levels after growth in 3% O_2_. Under those conditions the *Fmr1* mRNA in WT cells increased modestly, while the transcript in PM mESCs showed a larger increase, resulting in transcript levels that were 2.7-fold higher than in WT mESCs (Figure 5A). This suggests a heretofore unappreciated role of O_2_ tension in the regulation of the PM alleles. Note that neither the levels of the pluripotency markers Nanog, Oct4, Sox2, and Rex1 nor the repeat size were affected by O_2_ concentrations (Supplementary Figure S1). Despite the elevated level of *Fmr1* mRNA in the PM cells, western blots showed sharply reduced FMRP levels in these cells (Figure 5B) comparable to previous observations in mice brain (Entezam et al., 2007) and human cells (Kenneson et al., 2001).

### The 5′ End of the Mouse *Fmr1* Gene Also Forms a Stable R-Loop in mESCs

**FIGURE 6:**
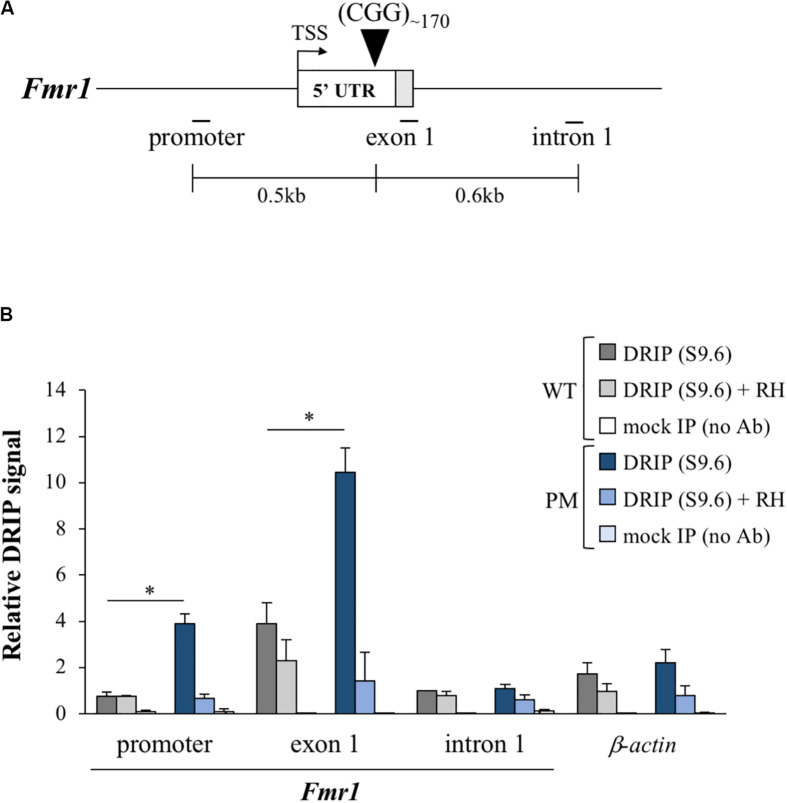
R-loop formation in the FX PM mESCs. **(A)** Diagram of the *Fmr1* gene 5′ region showing the relative positions of the qPCR amplicons used in DNA:RNA immunoprecipitation (DRIP) experiments. White box is exon 1 5′ UTR and gray the coding sequence (CDS). TSS is the transcriptional start site. Numbers indicate the distances from the CGG repeats. **(B)** R-loop enrichment in the *Fmr1* locus and β*-*actin (positive control gene; Skourti-Stathaki et al., 2019) was determined by DRIP analysis in 3 WT and 3 PM lines cultured at 3% O_2_. Pretreatment with RNase H (RH) was used as a control for the pull down. Values are relative to intron 1 in WT cells and displayed as mean ± SEM. *p < 0.01.

R-loops form on the 5′ end of the human *FMR1* gene where they have been shown to be enriched on expanded alleles compared to normal controls (Groh et al., 2014; Loomis et al., 2014; Kumari and Usdin, 2016; Abu Diab et al., 2018). Using DNA: RNA immunoprecipitation (DRIP) with an antibody (S9.6) that recognizes DNA:RNA hybrids (Boguslawski et al., 1986), we tested whether the *Fmr1* transcript also forms an R-loop in mESCs. We found that R-loops indeed form on the mouse *Fmr1* locus in both WT and PM mESCs (Figure 6). However, as in humans, R-loop levels in the PM cells were 5- and 2.7-fold higher than in WT in the promoter and exon 1 regions, respectively, (Figure 6B). Thus, these cells may be useful for understanding the factors that promote R-loop formation as well as some of its downstream consequences.

### The PM mESCs Also Show Elevated Mitochondrial Copy Numbers

**FIGURE 7:**
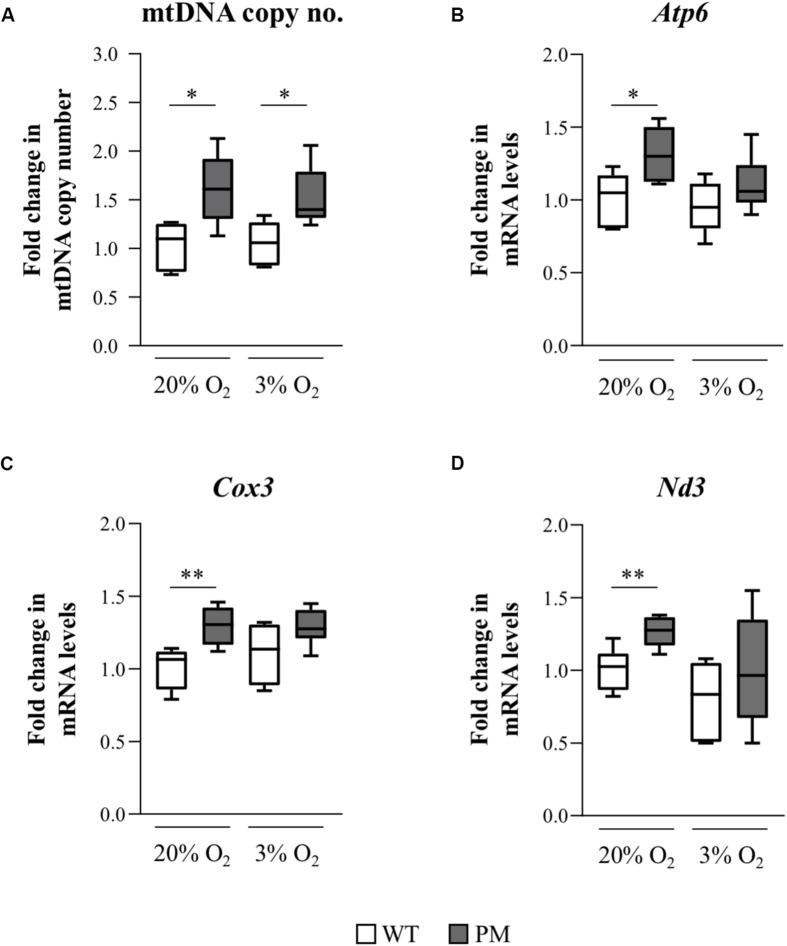
Mitochondrial DNA copy number and mitochondrial gene expression in FX PM mESCs. **(A)** mtDNA copy number was measured using qPCR to determine levels of a mitochondrial gene, *COXI*, relative to a nuclear gene, *GAPDH*, in 3 WT and 3 PM lines cultured at 20% or 3% O_2_. Values are relative to the average value of WT cells cultured at 20% O_2_ and displayed as mean ± SEM. *n* = 5; *p < 0.03. **(B–D)** Quantitative reverse-transcription-PCR analysis for the expression of the mitochondrial-encoded genes *Atp6* **(B)**, *Cox3* **(C)**, and *Nd3* **(D)** in 3 WT and 3 PM lines cultured at 20% or 3% O_2_ using β-actin for normalization. Values are relative to the average expression of the WT lines cultured at 20% O_2_ and shown as mean ± SEM. *n* = 6; *p < 0.02; and **p ≤ 0.006.

We assessed the mtDNA copy number in WT and PM mESCs grown at atmospheric and physiological O_2_ concentrations. PM mESCs showed significantly elevated mtDNA copy numbers compared to the WT at both O_2_ concentrations (Figure 7A). This was associated with a similar increase in expression of transcripts for three mitochondrially encoded genes, *Atp6*, *Cox3*, and *Nd3* (Figures 7B–D). Since elevated mtDNA copy number can be a response to increased oxidative stress (Malik and Czajka, 2013), it suggests that the mESCs may also be useful for studying the molecular basis of the mitochondrial changes and oxidative stress seen in PM carriers and PM mouse models (Ross-Inta et al., 2010; Conca Dioguardi et al., 2016; Napoli et al., 2016; Alvarez-Mora et al., 2017; Gohel et al., 2019).

## Discussion

Here we report the establishment of FX KI mESC lines that show a high frequency of progressive CGG repeat expansions in tissue culture. These expansions resemble those seen in the blood and saliva of human PM carriers in that all the repeat PCR profiles are consistent with a high frequency of expansions most of which involve the addition of 1–2 repeats [Figures 1–3 and (Zhao et al., 2019)]. Expansions in the mESCs also show a dependence on MSH2 (Figure 3) consistent with data from GWA studies that implicate MMR in the REDs (Bettencourt et al., 2016; Morales et al., 2016; Moss et al., 2017; Flower et al., 2019). The fact that in the 182 and 292 repeat lines expansion occurs in most cells in the population every few days (Figure 1B) indicates that the underlying trigger for expansion must be very common. It has been suggested that the repair of 8-oxoguanine generated by oxidative stress is this trigger (Kovtun et al., 2007). However, given that the total number of oxidative hits to DNA is thought to be of the order of 10^4^–10^5^ per cell per day in mammals (Ames et al., 1993), this would require the CGG-repeat tract in the mouse *Fmr1* locus to be orders of magnitude more prone to oxidative damage than the rest of the genome. Thus, it may be that oxidative damage is not the only trigger for expansions in the FXDs.

While most of the expansions we observe in these cell lines are small, their frequency makes it plausible for the large expansions, characteristic of the intergenerational transmission of FX alleles, to arise over time in the oocyte/embryo by the cumulative effect of these small expansions with or without a contribution from the rarer large expansions that are also seen (Figure 2). It is noteworthy that expansions are not seen in FX hESC lines, cells that correspond to a slightly later developmental stage than mESCs (Smith, 2017), nor human iPSC lines (Zhou et al., 2016), although expansions are seen in iPSCs from patients with Friedreich ataxia and Myotonic Dystrophy (Du et al., 2012, 2013). This suggests that very specific conditions are required for expansion at the FX locus that go beyond the simple requirement for genetic factors like MSH2 that are expressed at high levels in both ESCs and iPSCs (Du et al., 2012). Interestingly, the heart is an organ that does not show post-natal expansions in mice; however, the repeat PCR profiles in the heart suggest a very small number of pre-natal expansion events (Zhao et al., 2019). It is thus tempting to speculate that expansions occur in the early blastocyst, cease early on in the developing embryo and resume later in development only in expansion-prone tissues. Given that mESCs can be differentiated into different cell lineages, it should be possible to use these cells to test this hypothesis.

The fact that a significant number of expansions can be seen over weeks or even days in culture, means that these cells can be used to study some aspects of the expansion mechanism much more efficiently than is possible *in vivo*. An added advantage of these cells is that they consist of a single cell type, thus avoiding the problem of multiple cell types with different propensities to expand that is a confounding factor in different organs *in vivo* (Gazy et al., 2019; Zhao et al., 2020). Given the ease of gene editing in these cell lines, genetic factors that contribute to expansion risk can be rapidly identified. Furthermore, the presence of R-loops on the expanded CGG repeat region in the mESCs (Figure 6) is consistent with the hypothesis that R-loops play a role in repeat expansion (Schmidt and Pearson, 2016), an observation that can also be readily tested in these cells. However, not all factors that affect the extent of expansion may be apparent in these cells. For example, we show here that while the NHEJ protein LIG4 protects against expansion in mouse hepatocytes (Gazy et al., 2019), loss of LIG4 has little, if any effect in the mESCs. This is consistent with the idea that the extent of expansion that is seen in any particular cell type represents the balance between factors that promote expansion and those factors that protect against them. Thus, while factors that are required for expansion would be seen in all cells that expand including mESCs, factors that play a non-essential role in expansion or protect against expansion may not always be apparent in this cell type.

In addition to exhibiting repeat expansion, the PM mESCs also recapitulate the hyperexpression of the expanded *Fmr1* allele seen in mice and humans (Figure 5). Interestingly, we found that hyperexpression in these cells is exacerbated by growth at the low O_2_ concentrations that prevail *in vivo*. In this regard, it may be of interest that a number of proteins, including Egr-1 and Purα that bind CGG-DNA repeats (Cao et al., 1993; Weber et al., 2016), increase transcriptional activation of their target genes in response to reduced O_2_ tensions (Bae et al., 1999; Kong et al., 2007). We speculate that increased binding of such proteins to the long repeat tracts on PM alleles may contribute to *Fmr1* hyperexpression. In addition, R-loop formation has been suggested to promote gene expression by recruitment of activating chromatin modifiers. Thus, the mESC model may provide a simple system for testing these hypotheses.

In addition to the increase in *Fmr1* transcript levels and decrease in FMRP protein levels, an increase in mtDNA copy number and elevated transcripts from mitochondrially encoded genes was also observed (Figure 7). Mitochondrial dysfunction is thought to contribute to the pathology seen in human PM carriers where both increases and decreases in mtDNA copy number and mitochondrial activity have been reported (Ross-Inta et al., 2010; Napoli et al., 2016; Song et al., 2016; Loesch et al., 2017; Alvarez-Mora et al., 2019). The occurrence of both increased and decreased mtDNA copy numbers are not necessarily inconsistent. While an initial increase in mtDNA copy number is thought to reflect an adaptive response to mitochondrial dysfunction and the associated increased cellular stress (Lee et al., 2000; Malik and Czajka, 2013), over time the increased mtDNA copy number exacerbates chronic oxidative stress and mitochondrial damage and results ultimately in a reduction in the number of mitochondria (Lee et al., 2000; Malik and Czajka, 2013). Interestingly, in another RED, Huntington disease, a neurodegenerative condition that is associated with oxidative stress, a biphasic pattern of mtDNA copy number variation is seen, with increased copy numbers being seen prior to disease onset and decreased copy numbers thereafter (Petersen et al., 2014). Thus, the PM mESCs might represent early stages of response to cellular damage, preceding the decline in mtDNA copy number observed in some human PM brains (Alvarez-Mora et al., 2019). It is noteworthy that mitochondrial abnormalities are also seen in *Fmr1* knockout mice which do not express FMRP (D’Antoni et al., 2019; Shen et al., 2019). Hence, it remains to be seen whether the mitochondrial changes we observe are related to the expression of the PM allele, the deficiency of FMRP (Figure 5), or a combination of the two.

Current models for repeat-induced pathology in PM carriers include repeat-mediated sequestration of important CGG-binding proteins and toxic protein production by repeat-associated non-AUG (RAN) translation (Glineburg et al., 2018). Since these mESCs contain the endogenous murine stop codon situated immediately upstream of the repeats in exon 1 of the *Fmr1* gene, they do not produce high levels of FMRPolyG, the major product of RAN translation seen in humans (Todd et al., 2013). Thus, the mESCs we have derived can serve as a useful *in vitro* model system not only for modeling repeat expansion but also for studying the cellular abnormalities associated with the PM that may be independent of FMRPolyG.

## Data Availability Statement

All datasets generated for this study are included in the article/Supplementary Material.

## Ethics Statement

The animal study was reviewed and approved by NIDDK Animal Care and Use Committee.

## Author Contributions

IG and KU conceived the original idea and planned the experiments. IG, CM, and G-YK carried out the experiments. IG and KU wrote the manuscript with support from CM and G-YK. All authors contributed to the article and approved the submitted version.

## Conflict of Interest

The authors declare that the research was conducted in the absence of any commercial or financial relationships that could be construed as a potential conflict of interest.

## Footnotes

1 https://www.ncbi.nlm.nih.gov/gds

2 http://vassarstats.net/
