# Supplementary material for: CGG Repeat Expansion, and Elevated Fmr1 Transcription and Mitochondrial Copy Number in a New Fragile X PM Mouse Embryonic Stem Cell Model
Source: Front Cell Dev Biol. 2020 Jun 30;8:482. doi: 10.3389/fcell.2020.00482 (PMC7338602; doi:10.3389/fcell.2020.00482)
Supplement: Supplementary file 1 [file Data_Sheet_1.zip › Data Sheet 1/data sheet 1.pdf]

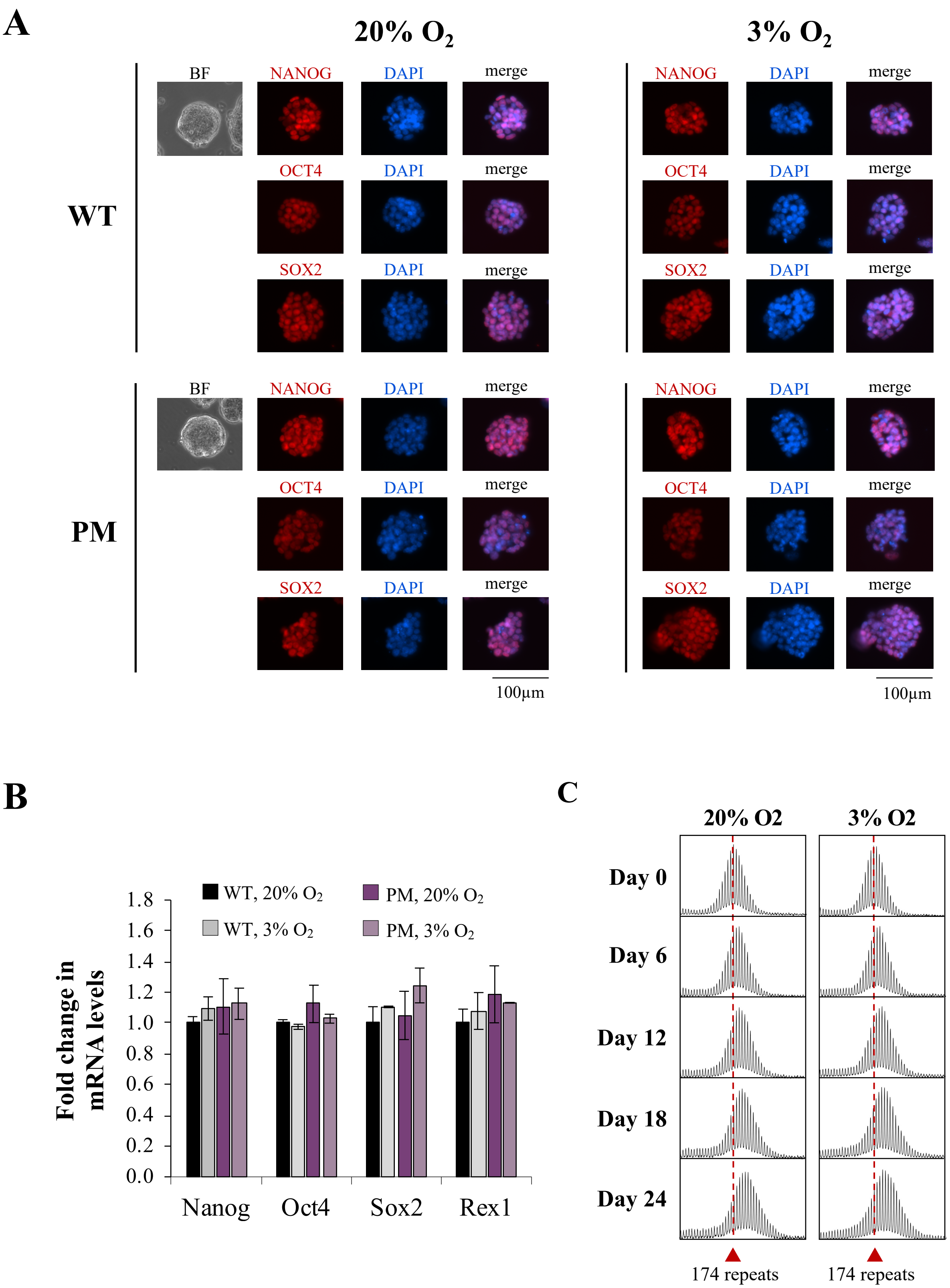

A
20% O_2
3% O_2
BF
NANOG
DAPI
merge
OCT4
SOX2
WT
PM
100μm
B
Fold change in mRNA levels
WT, 20% O_2
PM, 20% O_2
WT, 3% O_2
PM, 3% O_2
0.0
0.2
0.4
0.6
0.8
1.0
1.2
1.4
1.6
1.8
Nanog
Oct4
Sox2
Rex1
C
20% O2
3% O2
Day 0
Day 6
Day 12
Day 18
Day 24
174 repeats

Fig. 1S

Supplementary Figure 1. Oxygen concentration does not affect pluripotency markers or repeat instability. A) Brightfield image (BF) and immunofluorescent staining of mESC clones demonstrates that the mESCs have characteristic mESC-colony morphology and are positive for NANOG, OCT4 and SOX2 pluripotency markers. B) Quantitative reverse-transcription-PCR analysis for the expression of the ESC pluripotency markers *Nanog*, *Oct4*, *Sox2* and *Rex1* in 2 WT and 2 PM lines using *β-actin* for normalization. Values are relative to the average expression of the WT lines cultured in 20% O_2 and displayed as mean ± SEM. C) Representative repeat PCR profiles of PM lines grown in culture in either 20% or 3% O_2 for the indicated time. Dashed red line represents the initial repeat size at day 0. No differences were observed in 3 different ESC lines.

A

Msh2 KO clones

clone 1

```
      WT CACAGAAAGAAAGAGAGCCGACTTTTCCACTAAAGACATTTATCAGGATCTC-AACCGGTTACTGAAAGGCAAAAAAGGAGAACAGATAAATAGTGCTGC
Allele 1 CACAGAAAGAAAGAGAGCCGACTTTTCCACTAAAGACATTTATCAGGATCTCT-----------------------------------------------

      WT CCTACCAGAGATGGAGAATCAGgtctgtgggttacaaacatgtgcttaaatcactacagtgatgtttgtaaaggctaattcagtcactttaaaaattgca
Allele 1 ----------------------------------------------------------------------------------------------------

      WT tttatttattgtgtgcgcacacacccgtgctcatggacgcacaccaccgcgtgcgtgtggaagtgggaacaactttcgggagtttggtttttttcaccgt
Allele 1 --------------------------------------------------gtgcgtgtggaagtgggaacaactttcgggagtttggtttttttcaccgt

      WT gtgaaccctggggtaagcgcccaggcttggtgggagggtcttcatctgctgagcctcctcactggccacactcctgtcagattcactgtgaaatctgaac
Allele 1 gtgaaccctggggtaagcgcccaggcttggtgggagggtcttcatctgctgagcctcctcactggccacactcctgtcagattcactgtgaaatctgaac

      WT tctgcgtcggtgatactattgtacgtacaataaggtttgtgaaaaacaggaaagtgactatgcatggcagttaatcccagcactcgggaggcagaggcag
Allele 1 tctgcgtcggtgatactattgtacgtacaataaggtttgtgaaaaacaggaaagtgactatgcatggcagttaatcccagcactcgggaggcagaggcag
```

clone 2

```
      WT CACAGAAAGAAAGAGAGCCGACTTTTCCACTAAAGACATTTATCAGGATCTCAACCGGTTACTGAAAGGCAAAAAAGGAGAACAGATAAATAGTGCTGCC
Allele 1 CACAGAAAGAAAGAGAGCCGACTTTTCCACTAAAGACATTTATCAGGATCTC------------------------------------------------

      WT CTACCAGAGATGGAGAATCAGgtctgtgggttacaaacatgtgcttaaatcactacagtgatgtttgtaaaggctaattcagtcactttaaaaattgcat
Allele 1 ----------------------------------------------------------------------------------------------------

      WT ttatttattgtgtgcgcacacacccgtgctcatggacgcacaccaccgcgtgcgtgtggaagtgggaacaactttcgggagtttggtttttttcaccgtg
Allele 1 ------------------------------------------------cgtgcgtgtggaaatgggaacaactttcgggagtttggtttttttcaccgtg

      WT tgaaccctggggtaagcgcccaggcttggtgggagggtcttcatctgctgagcctcctcactggccacactcctgtcagattcactgtgaaatctgaact
Allele 1 tgaaccctggggtaagcgcccaggcttggtgggagggtcttcatctgctgagcctcctcactggccacactcctgtcagattcactgtgaaatctgaact

      WT ctgcgtcggtgatactattgtacgtacaataaggtttgtgaaaaacaggaaagtgactatgcatggcagttaatcccagcactcgggaggcagaggcagg
Allele 1 ctgcgtcggtgatactattgtacgtacaataaggtttgtgaaaaacaggaaagtgactatgcatggcagttaatcccagcactcgggaggcagaggcagg
```

B

Lig4 KO clones

clone 1

```
      WT AGGATGGCTTATGGAATCAAAGAAACCATGCTCGCTAAGCTCTACATCGAATTGCTGAACTTACCACGAGAAGGCAAGGATGCCCAGAAGCTCCTCAATT
Allele 1 AGGATGGCTTATGGAATCAAAGAAACCATGCTCGCTAAGCTCTACATCGAATTGCTGAACTTACCACGAGAAGGCAAGGATGCCCAGAAGCTCCTCAATT
Allele 2 AGGATGGCTTATGGAATCAAAGAAACCATGCTCGCTAAGCTCTACATCGAAT------------------------------------------------

      WT ACCGAACCCCCAGTGGAGCTCGCACGGATGCTGGGGACTTTGCCATGATTGCATACTTTGTTTTGAAGCCAAGGTGCTTACAGAAAGGAAGCTTAACCAT
Allele 1 ACCGAACCCCCAGTGGAGCTCGCACGGATGCTGGGGACTTTGCCATGATTGCATACTTTGTTTTGAAGCCAAGGTGCTTACAGAAAGGAAGCTTAACCAT
Allele 2 ACCGAACCCCCAGTGGAGCTCGCACGGATGCTGGGGACTTTGCCATGATTGCATACTTTGTTTTGAAGCCAAGGTGCTTACAGAA---------------

      WT ACAGCAGGTAAATGAACTCTTAGACTTAGTTGCCAGCAATAACTCTGGCAAAAAAAAAGACCTAGTGAAAAAGAGCCTTCTTCA-GTTAATAACCCAGAG
Allele 1 AC-------------------------------------------------------------------------------TCA-GTTAATAACCCAGAG
Allele 2 ------------------------------------------------------------------------------------gGTTAATAACCCAGAG

      WT TTCAGCACTGGAGCAAAAATGGCTGATTCGCATGATTATCAAAGACTTAAAGCTTGGCATCAGTCAGCAAACAATATTTTCCATTTTCCACAATGATGCA
Allele 1 TTCAGCACTGGAGCAAAAATGGCTGATTCGCATGATTATCAAAGACTTAAAGCTTGGCATCAGTCAGCAAACAATATTTTCCATTTTCCACAATGATGCA
Allele 2 TTCAGCACTGGAGCAAAAATGGCTGATTCGCATGATTATCAAAGACTTAAAGCTTGGCATCAGTCAGCAAACAATATTTTCCATTTTCCACAATGATGCA
```

clone 2

```
      WT AGGATGGCTTATGGAATCAAAGAAACCATGCTCGCTAAGCTCTACATCGAATTGCTGAACTTACCACGAGAAGGCAAGGATGCCCAGAAGCTCCTCAATT
Allele 1 AGGATGGCTTATGGAATCAAAGAAACCATGCTCGCTAAGCTCTACATCGAATTGCTGAACTTACCACGAGAAGGCAAGGATGCCCAGAAGCTCCTCAATT
Allele 2 AGGATGGCTTATGGAATCAAAGAAACCATGCTCGCTAAGCTCTACATCGAAT------------------------------------------------

      WT ACCGAACCCCCAGTGGAGCTCGCACGGATGCTGGGGACTTTGCCATGATTGCATACTTTGTTTTGAAGCCAAGGTGCTTACAGAAAGGAAGCTTAACCAT
Allele 1 ACCGAACCCCCAGTGGAGCTCGCACGGATGCTGGGGACTTTGCCATGATTGCATACTTTGTTTTGAAGCCAAGGTGCTTACAGAAAGGAAGCTTAACCAT
Allele 2 ----------------------------------------------------------------------------------------------------

      WT ACAGCAGGTAAATGAACTCTTAGACTTAGTTGCCAGCAATAACTCTGGCAAAAAAAAAGACCTAGTGAAAAAGAGCCTTCTTCAGTTAATAACCCAGAGT
Allele 1 AC-------------------------------------------------------------------------------TCAGTTAATAACCCAGAGT
Allele 2 ------------------------------------------------------------------------------------------AACCCAGAGT

      WT TCAGCACTGGAGCAAAAATGGCTGATTCGCATGATTATCAAAGACTTAAAGCTTGGCATCAGTCAGCAAACAATATTTTCCATTTTCCACAATGATGCAG
Allele 1 TCAGCACTGGAGCAAAAATGGCTGATTCGCATGATTATCAAAGACTTAAAGCTTGGCATCAGTCAGCAAACAATATTTTCCATTTTCCACAATGATGCAG
Allele 2 TCAGCACTGGAGCAAAAATGGCTGATTCGCATGATTATCAAAGACTTAAAGCTTGGCATCAGTCAGCAAACAATATTTTCCATTTTCCACAATGATGCAG
```

C

Prkdc KO clones

clone 1

**WT**	gtgtcactcccaagagagcagcatggatctgctggaggccaggactggaagttttgctgaaggggaggatatttttctatatttcctctttagaaaattg
**Allele 1**	gtgtcactcccaagagagcagcatggatctgctggaggccaggactggaagttttgctgaaggggaggatatttttctatatttcctctttagaaaattg
**Allele 2**	gtgtcactc---
**WT**	taatctgtttctttagTTACTTCAGATACTAAGAAGTACCAGACTCATGGATGAATTTAAGATTGGAGAATTATTTAACAAATTCTATGGAGAACTTGCA
**Allele 1**	taatctgtttctttagTTACTTCAGATACTAAGAAGTACCAGACTCATGGATGAATTTA---
**Allele 2**	--
**WT**	TCAAAATCAAAACTACCTGATACAGgtgagatgctattttcaaatattttaacaaattaaccttagctaacaatttcctcaacaaaggtat---------
**Allele 1**	--ctaacaatttcctcaacaaaggtat---------
**Allele 2**	---aacaagggt
**WT**	--tttgctgaaaaaaaattagcacaatggaaacaaaaggattccttaagagaaaacacataaagtatagcttagcttatagaagtagatttagataaaac
**Allele 1**	--tttgctgaaaaaaaat**tag**cacaatggaaacaaaaggattccttaagagaaaacacataaagtatagcttagcttatagaagtagatttagataaaac
**Allele 2**	actttgctgaaaaaaaattagcacaatggaaacaaaaggattccttaagagaaaacacataaagtatagcttagcttatagaagtagatttagataaaac

clone 2

**WT**	tgttgttggtctctgggaagttacagttatgtttagcctgccttttccctgtgatctcctactgatcactcaagccagactttcctataaccccaagtag
**Allele 1**	tgttgttggtctctgggaagttacagttatgtttagcctgccttttccctgtgatctcctactgatcactcaagccagactttcctataaccccaagtag
**Allele 2**	tgttgttggtctctgggaagttacagttatgttta---
**WT**	gtgtcactcccaagagagcagcatggatctgctggaggccaggactggaagttttgctgaaggggaggatatttttctatatttcctctttagaaaattg
**Allele 1**	gtgtcactcccaagagagcagcatggatctgctggaggccaggactggaagttttgctgaaggggaggatatttttctatatttcctctttagaaaattg
**Allele 2**	--
**WT**	taatctgtttctttagTTACTTCAGATACTAAGAAGTACCAGACTCATGGATGAATTTAAGATTGGAGAATTATTTAACAAATTCTATGGAGAACTTGCA
**Allele 1**	taatctgtttctttagTTACTTCAGATACTAAGAAGTACCAGACTCATGGATGAATTTAA--
**Allele 2**	--
**WT**	TCAAAATCAAAACTACCTGATACAGgtgagatgctattttcaaatattttaacaaattaaccttagctaacaatttcctcaacaaaggtattttgctgaa
**Allele 1**	--c**taa**caatttcctcaacaaaggtattttgctgaa
**Allele 2**	--
**WT**	aaaaaattagcacaatggaaacaaaaggattccttaagagaaaacacataaagtatagcttagcttatagaagtagatttagataaaacttgggaagaat
**Allele 1**	aaaaaattagcacaatggaaacaaaaggattccttaagagaaaacacataaagtatagcttagcttatagaagtagatttagataaaacttgggaagaat
**Allele 2**	--
**WT**	tttatgaaaacctaggaattttgtgttttattataaaattacttaaaacattgcttaagctgggcgtggtggcacacacccttaatcccagcactcagga
**Allele 1**	tttatgaaaacctaggaattttgtgttttattataaaattacttaaaacattgcttaagctgggcgtggtggcacacacccttaatcccagcactcagga
**Allele 2**	--
**WT**	ggcagaggcaggcggatttctgagttcgaggccagcctggtctacaaagtgagttccaggacagccagggctatacagagaaaccgtgtctcgaaaaaaa
**Allele 1**	ggcagaggcaggcggatttctgagttcgaggccagcctggtctacaaagtgagttccaggacagccagggctatacagagaaaccgtgtctcgaaaaaaa
**Allele 2**	--
**WT**	aagaaaaacaaacaaacaaacaaaaaactaaccaaacaacaaaacaaacaaacaaacaaaacattgcttaaacatgcttgtatatgtgcaggtgcatgtg
**Allele 1**	aagaaaaacaaacaaacaaacaaaaaactaaccaaacaacaaaacaaacaaacaaacaaaacattgcttaaacatgcttgtatatgtgcaggtgcatgtg
**Allele 2**	--
**WT**	tgtgaagtcataggacagttgtagagtcagttctcttcttgtaccatggaggttcagggactcagggcttgacagcaagtgcctgtaaagtgctcccctg
**Allele 1**	tgtgaagtcataggacagttgtagagtcagttctcttcttgtaccatggaggttcagggactcagggcttgacagcaagtgcctgtaaagtgctcccctg
**Allele 2**	--
**WT**	tgctggctggctggtcctcaaaccatgttgaaccaaacatcctcttatttctcatttatacattttgttattttacttacttaaatgtagtttgctttc
**Allele 1**	tgctggctggctggtcctcaaaccatgttgaaccaaacatcctcttatttctcatttatacattttgttattttacttacttaaatgtagtttgctttc
**Allele 2**	--
**WT**	atttatctgatcttatttttcagTTTTAGAAAAAGTCTATGAGCTCCTGGGAGT
**Allele 1**	atttatctgatcttatttttcagTTTTAGAAAAAGTCTATGAGCTCCTGGGAGT
**Allele 2**	-tttatctggtcttatttttcagTTT**TAG**AAAAAGTCTATGAGCTCCTGGGAGT

Figure 2S: Characterization of CRISPR/Cas9 edited clones. Depiction of the WT sequence and 2 edited *Msh2* (A), *Lig4* (B) and *Prkdc* (C) clones. In all cases the text in bold indicates a stop codon and underlined text a point mutation. In the case of the *Msh2* cell lines no evidence was seen of a second allele (A). This would be consistent with either both alleles having the same gene edit or the presence of a large deletion in the second allele that eliminated one or both primer binding sites. Text in upper case indicate exons, exon 4 in the case of *Msh2*, exon 2 in the case of *Lig4* and exons 5 and 6 in the case of *Pkrdc* lines, with the text in lower case indicating the adjacent introns.

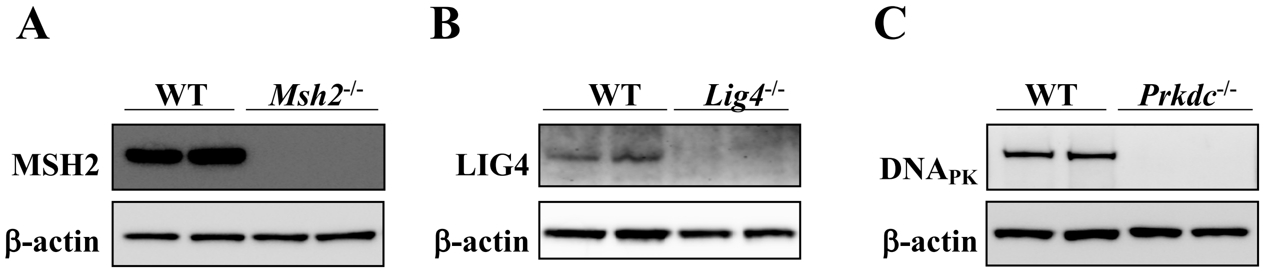

Figure 3S: Confirmation of successful CRISPR/Cas9 gene editing. A) Western blot analysis of MSH2 expression in 2 WT control and 2 *Msh2-/- Fmr1* PM lines shows no expression in 2 independent edited clones. B) Western blot analysis of LIG4 expression in 2 WT control and 2 *Lig4 -/- Fmr1* PM lines shows no expression in 2 independently edited clones. C) Western blot analysis of DNA-PK expression in 2 WT control and 2 *Prkdc*$^{-/-}$ mESCs shows no expression in 2 independent edited clones.
