# Supplementary material for: CGG Repeat Expansion, and Elevated Fmr1 Transcription and Mitochondrial Copy Number in a New Fragile X PM Mouse Embryonic Stem Cell Model
Source: Front Cell Dev Biol. 2020 Jun 30;8:482. doi: 10.3389/fcell.2020.00482 (PMC7338602; doi:10.3389/fcell.2020.00482)
Supplement: Supplementary file 2 [file Table_1.docx]

Supplementary Material

# Supplementary Tables

**Supplementary Table 1. CRISP-cas9 gRNAs and ssODNs sequences**

| **Name** | **Sequence** |
| --- | --- |
| MSH2 gRNA 1 | ACATTTATCAGGATCTCAAC |
| MSH2 gRNA 2 | CCACTTCCACACGCACGCGG |
| MSH2 ssODN | TGATCACAGAAAGAAAGAGAGCCGACTTTTCCACTAAAGACATTTATCATGTGGAAGTGGGAACAACTTTCGGGAGTTTGGTTTTTTTCACCGTGTGAAC |
| LIG4 gRNA 1 | AGGAAGCTTAACCATACAGC |
| LIG4 gRNA 2 | TCTGGGTTATTAACTGAAGA |
| LIG4 ssODN | CATGATTGCATACTTTGTTTTGAAGCCAAGGTGCTTACAGAAAGGAAGCTAATAACCCAGAGTTCAGCACTGGAGCAAAAATGGCTGATTCGCATGATT |
| Prkdc gRNA 1 | CTCATGGATGAATTTAAGAT |
| Prkdc gRNA 2 | GTTGAGGAAATTGTTAGCTA |
| Prkdc ssODN | TCTGTTTCTTTAGTTACTTCAGATACTAAGAAGTACCAGACTCATGGATGAATTTTCCTCAACAAAGGTATTTTGCTGAAAAAAAATTAGCACAATGGAA |

**Supplementary Table 2. Primers**

| **Primers for confirmation of CRISPR-Cas9 edited clones:** | |
| --- | --- |
| Msh2-F | TGGGAGAGGAGTCAGTGCTT |
| Msh2-R | GGTGGCTTTCATCCACTTGT |
| LIG4-F | CAGGACCAGAAGGAAAGCAG |
| LIG4-R | TGGCTTGTAAATGGACAGAGG |
| Prkdc-F | ATCAGCTGGCGTTTTGTTCT |
| Prkdc-R | CAAAGCCTGGCAGTACCTTC |
| **Primers for CGG repeat number determination:** | |
| FraxM4 | CTTGAGGCCCAGCCGCCGTCGGCC |
| FraxM5 | CGGGGGGCGTGCGGTAACGGCCCAA |
| FraxC | GCTCAGCTCCGTTTCGGTTTCACTTCCGGT |
| FraxF | GCCCCGCACTTCCACCACCAGCTCCTCCA |
| **Primers for DRIP:** | |
| Fmr1 promoter F | CGACTTGCAGTTCAAACAGG |
| Fmr1 promoter R | GGAAAGCGTAAAGGGCAGTA |
| Fmr1 exon 1 F | AGGACGGACGAGAAGATGGA |
| Fmr1 exon 1 R | GTACCTTGTAGAAAGCGCCATTGGAG |
| Fmr1 intron 1 F | GGCCGTTAGGAGGTTTGG |
| Fmr1 intron 1 R | GGGGAGCTCTCCAAAGTCTG |
| β−actin F | GAGGGGAGAGGGGGTAAA |
| β-actin R | GAAGCTGTGCTCGCGG |
| **Primers for mtDNA copy number evaluation:** | |
| COXI F | CTGAGCGGGAATAGTGGGTA |
| COXI R | TGGGGCTCCGATTATTAGTG |
| GAPDH int2 F | ATCCTGTAGGCCAGGTGATG |
| GAPDH int2 R | AGGCTCAAGGGCTTTTAAGG |

**Table S3. TaqMan assays**

| **Gene** | **Label** | **Thermo Fisher Scientific cat no.** |
| --- | --- | --- |
| Fmr1 | FAM | Mm01339582_m1 |
| Atp6 | FAM | Mm03649417_g1 |
| Nd3 | FAM | Mm04225292_g1 |
| Cox3 | FAM | Mm04225261_g1 |
| β-actin | VIC | 4352341E |

**Table S4. Primary antibodies**

| **Primary antibody** | **Antibody Details** | **Dilution factor** |
| --- | --- | --- |
| **Antibodies for Immunostaining** | | |
| anti-Nanog | NB100-588, Novus Biologicals | 1:100 |
| anti-Oct4 | sc-5279, Santa Cruz Biotech | 1:100 |
| anti-Sox2 | AB5603, Millipore-Sigma | 1:500 |
| **Antibodies for Western Blotting** | | |
| anti-MSH2 | ab70270, Abcam | 1:10,000 |
| anti-DNA_PK_ | sc-9051, Santa Cruz Biotech | 1:200 |
| anti-DNA Ligase IV | sc-271299, Santa Cruz Biotech | 1:200 |
| anti-FMRP | ab17722, Abcam | 1:900 |
| anti-β-actin | ab8227, Abcam | 1:10,000 |
